# Mononuclear and Tetranuclear Copper(II) Complexes Bearing Amino Acid Schiff Base Ligands: Structural Characterization and Catalytic Applications

**DOI:** 10.3390/molecules26237301

**Published:** 2021-12-01

**Authors:** Karla-Alejandra López-Gastélum, Enrique F. Velázquez-Contreras, Juventino J. García, Marcos Flores-Alamo, Gerardo Aguirre, Daniel Chávez-Velasco, Jayanthi Narayanan, Fernando Rocha-Alonzo

**Affiliations:** 1 Departamento de Investigación en Polímeros y Materiales, Universidad de Sonora, Rosales and Luis Encinas s/n, Col. Centro, Hermosillo 83000, Sonora, Mexico; 2 Facultad de Química, Universidad Nacional Autónoma de México, Circuito Exterior Cd. Universitaria, Coyoacán, Ciudad de México 04510, Ciudad de México, Mexico; juvent@unam.mx (J.J.G.); mfa@unam.mx (M.F.-A.); 3 Centro de Graduados e Investigación, Instituto Tecnológico de Tijuana, Apartado Postal 1166, Tijuana 22000, Baja California, Mexico; gaguirre777@gmail.com (G.A.); dchavez@hotmail.com (D.C.-V.); 4 División de Ingeniería en Nanotecnología, Universidad Politécnica del Valle de México, Av. Mexiquense s/n esquina Av. Universidad Politécnica, Col. Villa Esmeralda, Tultitlan 54910, Estado de México, Mexico; jnarayanan@upvm.edu.mx; 5 Departamento de Ciencias Químico Biológicas, Universidad de Sonora, Calle Rosales y Blvd. Luis Encinas s/n, Col. Centro, Hermosillo 83000, Sonora, Mexico

**Keywords:** copper complex, polynuclear complex, metallocyclic cyclopropanation, amino acid ligand, glycine ligand

## Abstract

Two new glycine-Schiff base copper(II) complexes were synthesized. Single crystal X-ray diffraction (SCXRD) allowed us to establish the structure of both complexes in the solid state. The glycine-Schiff base copper(II) complex derived from 2′-hydroxy-5′-nitroacetophenone showed a mononuclear hydrated structure, in which the Schiff base acted as a tridentate ligand, and the glycine-Schiff base copper(II) complex derived from 2′-hydroxy-5′-methylacetophenone showed a less common tetranuclear anhydrous metallocyclic structure, in which the Schiff base acted as a tetradentate ligand. In both compounds, copper(II) had a tetracoordinated square planar geometry. The results of vibrational, electronic, and paramagnetic spectroscopies, as well as thermal analysis, were consistent with the crystal structures. Both complexes were evaluated as catalysts in the olefin cyclopropanation by carbene transference, and both led to very high diastereoselectivity (greater than 98%).

## 1. Introduction

Schiff bases and their metallic complexes are compounds with a wide range of applications in the food and dye industry, analytical chemistry, catalysis, and agrochemicals [1,2]. Furthermore, these compounds have been shown to possess important biological activities such as anti-inflammatory, antibacterial, antifungal, antiparasitic, antiproliferative, and antiviral (including anti-HIV and anti-covid) properties [3,4]. Schiff’s base complexes are considered among the most critical stereochemical models in main group chemistry, and the coordination of transition metals due to their preparative accessibility and structural broadness [5]. Since Schiff bases are distinguished by their simple and easy synthesis; this fact and their capability to form complexes with different metals have caused Schiff bases to be considered as “privileged ligands” [6,7]. In this respect, the use of amino acids, in combination with a salicylaldehyde-type carbonyl source, has allowed the preparation of a very good chelating Schiff base family [8,9,10,11,12]. These ligands primarily act in bi and tridentate mode; reports of tetradentate mode are scarce [13]. Thus, interested in exploring the chelating capability of these kinds of ligands, we have synthesized two copper (II) complexes with glycine-Schiff base ligands. The first one has a common structure where the Schiff base acts as a tridentate binder, and a solvent molecule completes the coordination sphere, and the second one has a tetradentate coordination mode, forming a tetranuclear complex. On the other hand, we were also interested in exploring the catalytic applications of these metallic complexes, and, for this purpose, we selected the cyclopropanation of styrene. Cyclopropanation of olefins is of particular interest as it provides access to conformationally constrained and stereochemically rich cyclopropane rings, which represent key building blocks for the discovery and development of drugs and other biologically active molecules [14]. Copper–Schiff base complexes are of particular significance because they have proven to be effective for the intermolecular cyclopropanation of olefins as well as for intramolecular cyclopropanation; the successful industrial application of these kinds of catalysts makes them a significant achievement in catalysis [15]. Li et al. has pointed out that modifications of the salicylaldehyde moiety to improve the catalysts have not been reported so far; they studied the electronic effects of substituents on the salicylaldehyde framework and found that electron withdrawing groups, such as the nitro group, and electron donating groups, such as the methyl group, can influence the catalytic process [16]. Thus, we decided to evaluate the complexes synthesized by this type of catalysis.

## 2. Results and Discussion

### 2.1. Synthesis of Schiff Bases Ligands and Copper (II) Complexes

**Scheme 1:**
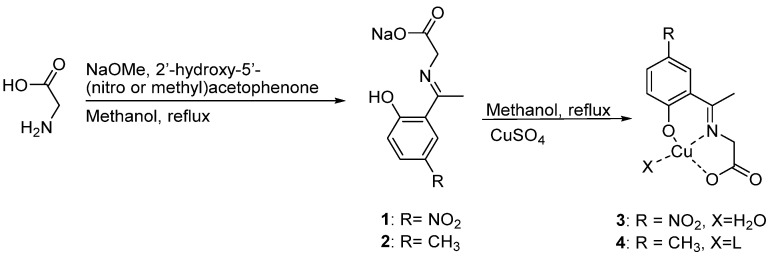
Synthesis of the copper (II) complexes.

The copper(II) complexes were prepared using a one-pot synthesis strategy based on a previously reported methodology [17]; the complete process is shown in Scheme 1. By glycine condensation with 2′-hydroxy-5′-nitroacetophenone and 2′-hydroxy-5′-methylacetophenone, **1** and **2** Schiff base ligands were achieved, respectively, in a 1:1 molar ratio. An intense yellow color was obtained for both ligands, which is characteristic of this kind of compound [18]. The reaction of copper (II) sulfate with the synthesized ligands led to the formation of the **3** and **4** complexes. The appearance of a dark green color was the first evidence of complex formation; these color changes are like those reported by other authors [19]. Copper (II) complexes characterization includes X-ray diffraction, spectroscopic and thermogravimetric analysis and is discussed below.

### 2.2. Single Crystal X-ray Diffraction (SCXRD)

Crystals of both complexes suitable for X-ray diffraction were obtained. The crystallographic analysis showed two new structures: **3** showed a monomeric structure, while **4** showed a tetrameric structure. A summary of crystallographic data is presented in the supporting information.

**Figure 1:**
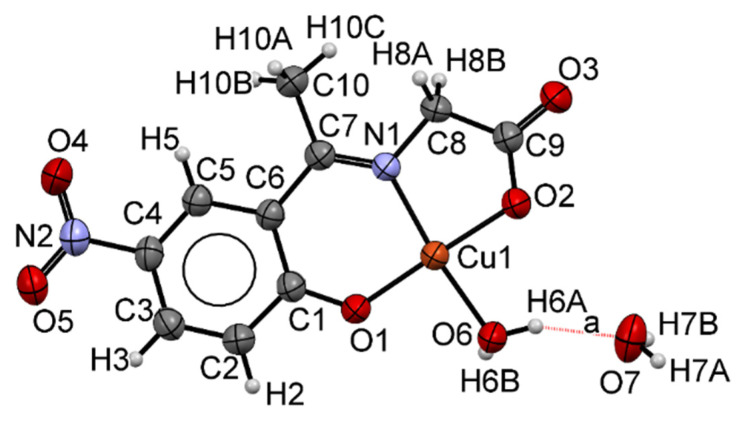
ORTEP drawing of complex **3** showing the atom numbering scheme and the thermal motion ellipsoids (50% probability level) for the non-hydrogen atoms.

The complex **3** structure with an atom labeling scheme is shown in Figure 1, which crystallizes in a tetragonal I4_1/a_ space group. The ORTEP diagram shows each copper (II) ion having a tetra-coordinate square planar geometry. Schiff base **1** acts as a tridentate ligand due to the imine nitrogen atom (N1), the phenolic oxygen atom (O1), and one of the carboxylate oxygen atoms (O2); the fourth donor atom comes from a water molecule (O6). The crystalline structure is stabilized by hydrogen bonds of the O–H ··· O type, which join water molecules [20]. Table 1 shows selected bond lengths and angles for complex **3**. The bond lengths of (Cu1)–(O1) [1.851(2) Å] and (Cu1)–(N1) [1.929(3) Å], (Cu1)–(O2) [1.902(2) Å] and (Cu1)–(O6) [1.927(2) Å] agree with tridentate dianionic Schiff base ligands [8,21].

The sum ∑ of the six inter-bond angles for complex **3** is 705.69°, which deviates from the ideal angle of 720°, suggesting that the geometry around the copper (II) ion is distorted square-planar. The irregularity in the square planar coordination is mainly due to the different bond angles around the copper (II) atom, ranging from 86.64 (11) to 95.28 (11)°. The two trans angles also deviate from the ideal values of a square planar geometry, viz. 176.10 (12) and 169.41 (12)°, possibly due to the chelation effect [22]. The C7=N1 [1.291(4) Å] bond in the molecular structure of **3** is longer than the typical value for C=N double bond (1.279 Å); the C9−O2 (Cu) [1.271 (4) Å] bond is shorter than the typical value for the carboxylic C−O bond (1.308 Å), but it is longer than a delocalized double bond in carboxylate anions (1.254 Å). The C9=O3 [1.237 (4) Å] bond is shorter than a delocalized double bond in carboxylate anions but longer than a carboxylic acid C=O bond (1.214 Å) [23].

**Figure 2:**
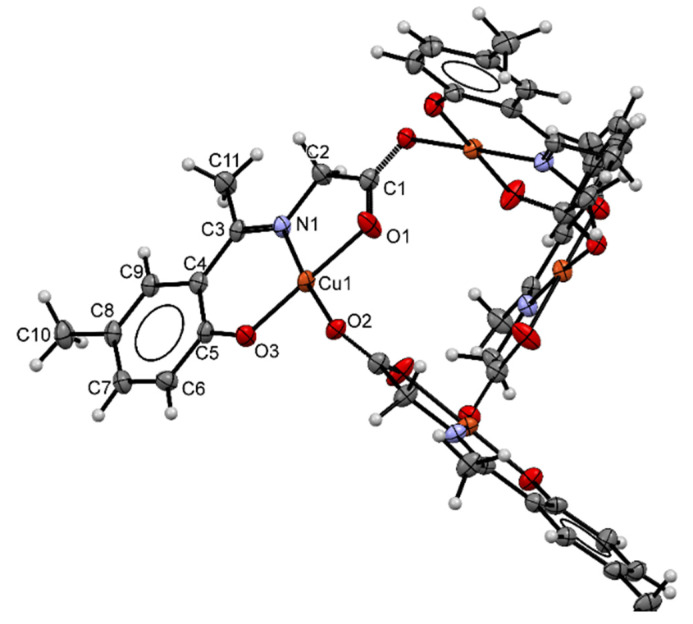
ORTEP drawing of complex **4** showing the atom numbering scheme and the thermal motion ellipsoids (50% probability level) for the non-hydrogen atoms.

Complex **4** with an atom labeling scheme is shown in Figure 2, which crystallizes in tetragonal space group I4_1/a_; the ORTEP diagram shows a cyclic tetrameric structure comprised of four copper(II) ions bonded to four ligand molecules in which each metal center has a distorted square planar environment. Metallocyclic structures are not common for amino acid Schiff base ligands. This is one of the few reports of this kind of structure [24]. Copper(II) centers are attached to two ligand units: the first ligand (**2**) coordinates the copper(II) ion through the imine nitrogen atom (N1), the phenolic oxygen atom (O3) and one of the carboxylate oxygen atoms (O1); a second ligand (2 *) completes the coordination sphere through one of the carboxylate oxygen atoms (O2 *) and acts as a tridentate ligand, coordinating a second copper(II) center through N1 *, O3 *, and O1 *, atoms; this pattern continues until a four-metal cycle is completed. Table 1 shows selected bond lengths and angles for complex **4**. The bond lengths of (Cu1)–(O1) [1.9467 (17) Å] and (Cu1)–(N1) [1.9288 (18) Å], (Cu1)–(O3) [1.8560(17) Å] and (Cu1)–(O2*) [1.9572 (15) Å] agree with values in related complexes with tridentate dianionic Schiff base ligands [25,26].

The sum ∑ of the six inter-bond angles for complex **4** is 712.08°, which deviates from the ideal angle of 720°, suggesting that the geometry around the copper(II) ion is distorted square-planar. The irregularity of the square planar coordination is mainly due to the different bond angles around the Cu atom, ranging from 85.82 (7) to 95.19 (7)°. The two *trans* angles also deviate from the ideal values of a square planar geometry, viz. 178.97 (7) and 173.12 (7)°, possibly due to the chelation effect [22,27]. In the molecular structure of **4**, the C3=N1 [1.298 (3) Å] bond is of a similar size to the one for complex **3**; the C1-O1 (Cu) [1.266 (3) Å] bond is slightly shorter than the same bond for complex **3**. Interestingly, the C1 *-O2 *(Cu) bond [1.246 (3) Å], which comes from the ligand unit that carries only one donor atom to the chelated metal center (which would correspond to C=O in 3), is shorter than the C1-O1(Cu) bond and slightly longer than the carbonyl bond in **3** [23].

### 2.3. FTIR Spectra Analysis of Schiff Bases Ligands and Copper (II) Complexes

IR spectroscopy has proven to be a suitable technique to elucidate the metal-ligand bonding mode. The coordinating atoms are determined by comparing the IR spectra of the free and complexed ligands. The significant IR data are given in Table 2. The IR spectrum of the ligands **1** and **2** shows bands at 3400 and 3239 cm^−1^ respectively, due to the ν(O-H) group of phenolic oxygen, and a strong band at 1616–1618 cm^−1^ due to azomethine stretching vibration ν(C=N) and carboxylate (C=O) [28,29,30]. The IR band due to the azomethine (C=N) and carboxylate (C=O) groups shifts to a lower frequency (13–15 cm^−1^) after complexing with a metal center; another change observed was the disappearance of the vibration ν(O-H) of the phenolic moiety at 3239–3400 cm^−1^, in agreement with the replacement of the hydrogen by the copper (II) ion [31]. Complex **3** shows a new broad band at 3100–3300 cm^−1^ and two weaker bands in the region of 755 and 733 cm^−1^ due to ν(–OH) rocking and wagging modes of vibrations, respectively, in agreement with the presence of a coordinated water molecule in the complex [32,33]. On the other hand, the absence of these water signals in complex **4** reveals an anhydride structure.

### 2.4. Thermal Analysis of the Copper (II) Complexes

**Figure 3:**
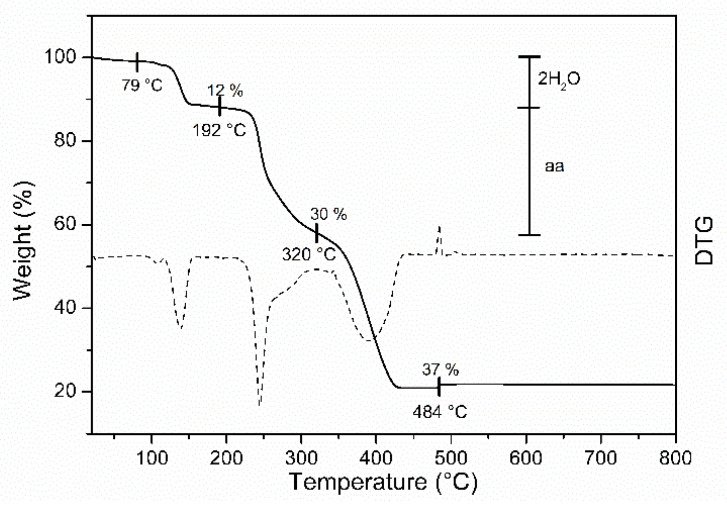
Thermogravimetric (TG/DTG) curves of **3**.

**Figure 4:**
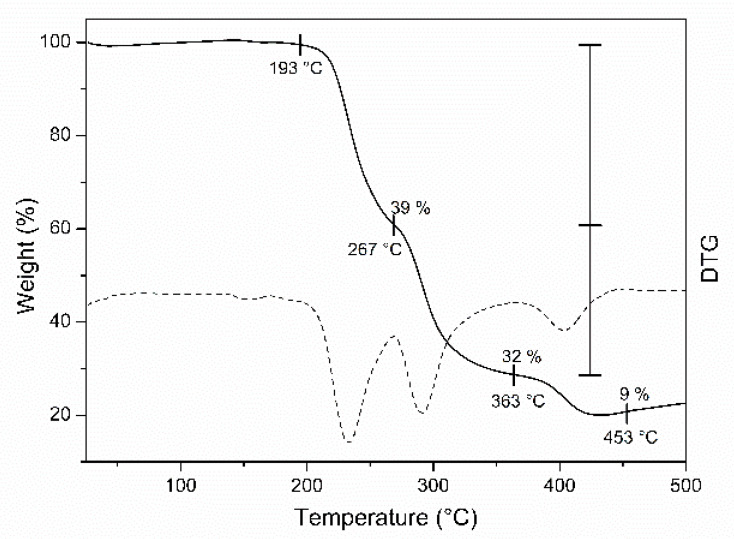
Thermogravimetric (TG/DTG) curves of **4**.

The TGA and DTG of the copper complexes are given in Figure 3 and Figure 4, and the thermal stability data are listed in Table 3. TGA data indicated that complex decomposition proceeds in two or three stages. Complex **3** shows a weight loss of 12 % at 79–192 °C, stage I, which corresponds to the removal of two water molecules from the complex and thus confirms the presence of coordinated water molecules inside the sphere [31,34,35,36]. In stage II, carried out at 192–320 °C, the evaporation of the organic amino acid moiety takes place. At 328–632 °C, the removal of another organic moiety from 5′-nitro-2′-hydroxyacetophenone is shown, stage III; and as a final stage, the air-stable metal oxide is left as a residue [37,38]. Complex **4** is decomposed into three stages. Stage I was found at 193–267 °C with a weight loss of 39%, maybe due to the loss of the amino acid moiety. Stage II, found at 267–363 °C and with a weight loss of 39%, was assigned to the removal of the 5′-methyl-2′-hydroxyacetophenone moiety, and stage III, where the air-stable metal oxide is left as a residue [39,40]. There was no weight loss observed for **4** in the 79–192 °C range, confirming the absence of water molecules in the coordination sphere.

### 2.5. UV-Vis Spectroscopy of Schiff Bases Ligands and Copper (II) Complexes

**Figure 5:**
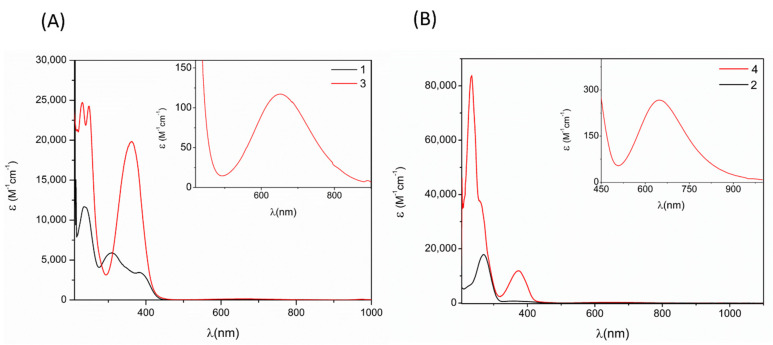
UV-Vis spectra of **1** and **3** (**A**), and **2** and **4** (**B**).

Figure 5 shows the absorption spectra of copper (II) complexes and free ligands recorded in methanol, and electronic spectral data are summarized in Table 4; free ligands have two absorption bands at 241–264 and 338–382 nm due to π → π * transitions of the aromatic ring and *n*–π * transitions of the C=N groups, respectively; the width of the second band reaches the violet region in the visible spectrum, resulting in the characteristic yellow complementary color [41]. The copper (II) complex spectra show the same π–π * and *n*–π * transitions, with a slight bathochromic shift; the permanence of these absorption bands is indicative of the presence of the ligand in the structure [42]. A broad and low energy band at ca. 645 is shown in both copper (II) complexes, which is attributed to the *d–d* transition (^2^ B1g-^2^ A1g); this is an important characteristic found in complexes in which the metallic center has an oxidation state of 2+ and a square-planar geometry [38,43].

### 2.6. Electron Paramagnetic Resonance of Copper (II) Complexes

Copper complexes EPR spectra in polycrystalline state at 298 K, and in methanol at 77 K, were recorded in the X band, using 100-kHz modulation frequency and 9.4 GHz microwave frequency. The spectral assignments are presented in Table 5 and the experimental and simulated best fits of the EPR spectra are given in Figures S3–S6 of the supplementary material.

The EPR spectra of **3** and **4** in the polycrystalline state at 298 K are of axial type giving rise to two g values, g|| and g ┴ with g_||_ > g_┴_ > g_e_ (2.0023), with no hyperfine lines in the parallel and perpendicular region, which is consistent with a d_x2-y2_ ground state in copper with an oxidation state of 2+ and a square planar geometry [44]. The variation in the g_||_ and g_┴_ values indicate that in the solid-state, the geometry of the compounds is affected by the nature of coordinating ligands. The geometric parameter G is calculated as G = g_||_ −2.0023/g _┴_ −2.0023 for axial spectra and it is a measure of the exchange interaction between copper centers in the polycrystalline compound. Accordingly, a parameter G greater than 4, indicates that exchange interaction may be negligible; however, a G value lower than 4 is congruent with a considerable exchange interaction in the solid complex [45,46,47]. The geometric parameter G for **3** and **4** are 4.4899 and 2.7323, respectively, which shows that the exchange interaction between the copper centers in the solid-state is insignificant for the first and strong for the second.

The EPR spectra recorded in a frozen state at 77 K gives more information about the complexes’ geometry. Both copper(II) complexes exhibit axial features [(g_||_ = 2.271, g_┴_ = 2.065) and (g_||_ = 2.266, g_┴_ = 2.053)] as well as well-resolved hyperfine splitting in parallel and perpendicular region due to the interaction of the odd electron with the nuclear spin (^63,65^ Cu, I = 3/2). For these tetracoordinate complexes **3** and **4**, the fact that g|| is greater than g_┴_ suggests a distorted square-planar structure consistent with the X-ray structural analysis [48].

### 2.7. Catalytic Activity of Copper (II) Complexes in Olefin Cyclopropanation

**Scheme 2:**
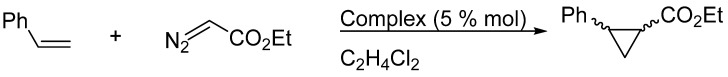
Cyclopropanation of styrene.

Carbene transference was used to assess the catalytic activity of both copper(II) complexes in olefin cyclopropanation. As a model reaction, we choose the formation of ethyl 2-phenylcyclopropane-1-carboxylate from styrene and the copper(II)-carbene complex formed in situ by the reaction of **3** or **4** with ethyl diazoacetate (EDA) (Scheme 2). The IR band at 2114 cm^−1^, due to the stretching of the N_2_ moiety in EDA, was used to follow the reaction progress; this signal was consumed entirely after 18 h of reaction in both complex catalytic evaluations. As a control in these catalytic evaluations, we used copper (II) sulfate; we found that after 160 h, the IR signal from the EDA had not completely disappeared. Carbene transfer can lead to two possible products, the trans and cis diastereomers; the comparison of the mass fragmentation patterns and the use of the NIST Standard Reference database, as well as the congruence of the retention times obtained and their comparison with those reported in the literature [49], allowed the diastereomers’ identification by GC-MS. The chromatogram was achieved using a chiral column (β™-DEX) and showed two peaks, at 13.28 and 13.48 min, for the cis isomer, and two peaks, at 12.63 and 12.90 min, for the trans isomer. On the other hand, the in situ formed carbene can react with the EDA, which is in excess in the reaction mixture, generating the homocoupling products: ethyl malate and ethyl fumarate. The presence of homocoupling products must be determined since their formation decreases the yield in the cyclopropane synthesis.

Results of cyclopropanation of styrene catalyzed by complexes **3** and **4** are presented in Table 6. As can be seen, the yields ranged from very good to excellent. The diastereoselectivity is quite high in both catalysts. Square-planar copper (II) complexes, with Schiff base ligands of similar structure of the ones presented in this work, have shown much lower trans/cis ratios. For example, in the complexes studied by Li et al., who used derivatives of salicylaldehyde and chiral alcohols in the formation of their Schiff bases, the trans/cis ratio was approximately 64:36 [16]; Iglesisas et al. used derivatives of salicylaldehyde and chiral amines obtained trans/cis ratios of 70:30 [50]; Maserson et al. synthesized complexes with Schiff bases derived from 2′-hydroxyacetophenone and paracyclophanyl, obtaining trans/cis ratios of 2:1 [51]. It does not seem to be influenced by the electronic groups in the salicylaldehyde moiety but it can be seen that complex **3**, with the nitro group as a substituent, forms a less homocoupling product than complex **4**, with the methyl group. Significant differences due to the polynuclear-cyclic structure of the complex **4** were not observed. One hypothesis is that during the carbene formation, the tetranuclear complex breaks its structure, generating a mononuclear carbene-complex, and this step could have a slight influence on the formation of the homocoupling product. These results encourage us to continue with these investigations; a more in-depth study will include changes in the substrate/catalyst ratio, as well as the evaluation with different substrates and at different temperatures [52,53,54].

## 3. Materials and Methods

### 3.1. General Procedure

All the reagents were purchased from Sigma Aldrich and used without any further purification. Infrared spectra (FTIR) were obtained using KBr pellets or NaCl windows on a Perkin-Elmer Frontier FT-IR/FIR spectrometer. Electronic spectra were recorded in methanol on a Perkin Elmer Lambda 20 UV–Vis spectrophotometer. Thermogravimetric analysis (TGA) was carried out with a Perkin-Elmer Pyris 1 TGA apparatus under an N_2_ atmosphere at a flow rate of 10 mL/min over a temperature range of 25 to 800 °C at a heating rate of 5 °C/min and analyzed using the Pyris version 11.1.1.0492 software package. Electron paramagnetic resonance spectra (EPR) of polycrystalline samples at 298 K and solution at 77 K were recorded in quartz tubes with a Joel JES-TE300 spectrometer equipped with a cylindrical cavity (TE011 mode) operating at X band frequency (9.4 GHz) at 100 KHz field modulation. Gas chromatograms were recorded on an Agilent 7890B-GC System equipped with a mass selective detector, the Agilent 5977A.

### 3.2. Synthesis of the Copper (II) Complexes

The copper (II) complexes were prepared using a one-pot synthesis strategy. 2′-hydroxy-5′-nitroacetophenone (181 mg, 1 mmoL), or 2′-hydroxy-5′-methylacetophenone (150 mg, 1 mmoL), dissolved in 10 mL of methanol, was added to a hot methanolic solution (20 mL) containing a mixture of sodium methoxide (54 mg, 1 mmoL) and glycine (75 mg, 1 mmoL). The reaction mixture was refluxed overnight, and the ligands formed in this step were named **1** and **2**, according to the starting ketones used. Copper sulfate pentahydrate (250 mg, 1 mmoL, in 10 mL of methanol) was added and the reflux was maintained overnight. The reaction mixture was cooled to room temperature and filtered to remove any precipitate. Suitable single crystals for structure determination by X-ray diffraction were obtained by slow evaporation of the mother liquors. The copper (II) complexes formed in this final step were named **3** and **4**, respectively. **3**. Yield: 95%; m.p. 217–218 °C. IR (KBr, cm^−1^): 3065br, 1601vs, 1433m, 1382m, 1484s, and 1324vs. Anal calc. for C_10_H_12_CuN_2_O_7_ (335.76): C, 35.62; H, 3.60; N, 8.34. Found: C, 35.62; H, 3.47; N, 8.77 %. **4**. Yield: 82 %; m.p. 233–235 °C. IR (KBr, cm^−1^): 1605vs, 1452m, 1305m (br, broad; vs, very strong; s, strong; m, medium). Anal calc. for C_44_H_44_Cu_4_N_4_O_12_ (1074.99): C, 49.16; H, 4.13; N, 5.21. Found: C, 50.44; H, 3.59; N, 4.40%.

### 3.3. X-ray Crystallography

A single crystal of **3** mounted on a glass fiber was studied with a SuperNova, Dual, Cu at zero, AtlasS2 diffractometer. The crystal was kept at 295.6 (2) K during data collection. The SHELXT [55] and SHELXL [56] software were used for structure solution and refinement, and the Olex2 [57] software was used to prepare material for publication. Full-matrix least-squares refinement was carried out by minimizing (Fo^2^–Fc^2^)^2^. A suitable single crystal of compound **4** was mounted on glass fiber, and crystallographic data were collected with an Oxford Diffraction Gemini “A” diffractometer with a CCD area detector at 130 K, with lMoKa = 0.71073. Unit cell parameters were determined with a set of three runs of 15 frames (1° in w). The double-pass method of scanning was used to exclude any noise [58]. The collected frames were integrated by using an orientation matrix determined from the narrow frame scans. Final cell constants were determined by a global refinement. Collected data were corrected for absorbance by using analytical numeric absorption correction using a multifaceted crystal model based on expressions upon the Laue symmetry with equivalent reflections [59]. Structure solution and refinement were carried out with the SHELXS-2018 [55] and SHELXL-2018 [56] packages. WinGX v2020.2 [60] software was used to prepare material for publication. Full-matrix least-squares refinement was carried out by minimizing (Fo^2^–Fc^2^)^2^. All non-hydrogen atoms were anisotropically refined H atoms attached to C atoms and were placed in geometrically idealized positions and refined as riding on their parent atoms, with C–H = 0.95–0.99 Å and with Uiso(H) = 1.2 Ueq(C) for aromatic and methylene groups, and Uiso(H) = 1.5Ueq(C) for the methyl group. Attempts made to model the solvent molecule were not successful; the SQUEEZE [61] option in PLATON indicated there was a solvent cavity of 67 Å^3^. In the final cycles of refinement, this contribution of 16 electrons to the electron density was removed from the observed data. For the electron density, the F (000) value in the molecular weight and the formula are given without considering the results obtained with SQUEEZE. The crystallographic data for the structure described in this paper have been deposited with the Cambridge Crystallographic Data Centre as supplementary publication no. CCDC 2,085,195 (for **3**) and 2,082,007 (for **4**). Copies of the data can be obtained free of charge on application to CCDC, 12 Union Road, Cambridge, CB2 1EZ, UK (fax: (+44) 1223-336-033, e-mail: deposit@ccdc.cam.ac.uk).

### 3.4. Catalytic Assay

Under nitrogen, ethyl diazoacetate (EDA) (114 mg, 1 mmoL, in 2.8 mL of 1,2-dichloroethane) was slowly added via a syringe pump (injection time: 6 h) to a mixture of the catalyst (0.05 mmoL, 5% moL), styrene (1 mL, 8.7 mmoL) and solvent (2 mL of 1,2-dichloroethane); the reaction was carried out at room temperature. The reaction was monitored by IR spectroscopy, following the EDA signal at 2114 cm^−1^. After complete EDA signal disappearance, the reaction mixture was stirred for a further 18 h to ensure complete reaction. The mixture was then purified through a short silica gel plug. After that, the solvent and unreacted styrene were eliminated under reduced pressure. Yield and diastereomeric ratio were determined by GC: column β™-DEX 120, 30 m X 0.25 mm X 0.25 μm; an initial oven temperature of 60 °C was held for 2 min, followed by a ramp of 15 °C/min to 210 °C, held for 10 min. Temperatures at the injection port and detector were kept constant at 210 °C. Total chromatographic separation was achieved in 22 min. The result is the mean of three independent experiments, each injected in triplicate. The same reaction was carried out using a copper (II) sulfate as “blank”; we found that the disappearance of the EDA IR signal took more than 160 h, and the coupling by-products were found in chromatography.

## 4. Conclusions

Two new glycine-Schiff base–copper (II) complexes were synthesized and their structures were determined by X-ray diffraction. Interestingly, it was found that one of these complexes presented an unusual structure in which the Schiff base acts as a tetradentate ligand, forming a cyclic–tetranuclear complex. The second complex has a monomeric structure, which is more common. The only difference between these two complexes is the nitro and the methyl substituent at the 5′ position in the starting ketone used for the formation of the Schiff bases; a possible reason for the structural differences could be related to this fact. Further research into how or why one structure is preferred over another is required. The spectroscopic studies, as well as the results of the thermal analysis, were consistent with the found structures. The results from catalytic evaluation were promising. Both copper (II) complexes led to a very high *trans/cis* ratio, which may be relevant for the design of new catalysts for different chemical transformations. However, there were no significant differences in the mononuclear or tetranuclear activity, which could suggest that, at least during the catalytic process, the carbene formation decomposes the tetranuclear structure, generating a mononuclear carbene complex.

## Figures and Tables

**Table 1:** Selected bond lengths (Å) and bond angles (°) for **3** and **4**.

3		4	
Cu (1)-O (1)	1.851 (4)	Cu (1)-O (3) # 1	1.8560 (17)
Cu (1)-N (1)	1.929 (3)	Cu (1)-N (1) # 1	1.9288 (18)
Cu (1)-O (2)	1.902 (2)	Cu (1)-O (1) # 1	1.9467 (17)
Cu (1)-O (6)	1.927 (2)	Cu (1)-O (2)	1.9572 (15)
O (1)-Cu (1)-N (1)	95.28 (11)	O (3) # 1-Cu (1)-N (1) # 1	95.19 (7)
O (1)-Cu (1)-O (2)	176.10 (12)	O (3) # 1-Cu (1)-O (1) # 1	178.97 (7)
N (1)-Cu (1)-O (2)	86.30 (11)	N (1) # 1-Cu (1)-O (1) # 1	85.82 (7)
O (1)-Cu (1)-O (6)	89.40 (10)	O (3) # 1-Cu (1)-O (2)	91.42 (7)
N (1)-Cu (1)-O (6)	169.41 (12)	N (1) # 1-Cu (1)-O (2)	173.12 (7)
O (2)-Cu (1)-O (6)	89.64 (11)	O (1) # 1-Cu (1)-O (2)	87.56 (7)

**Table 2:** Infrared spectral data (cm^−1^) of Schiff base ligands and their complexes.

Compound	1	3	2	4
V (OH)	3400	3065	3239	-
v (C=O)	1616	1601	1618	1605
v (C=N)	1616	1601	1618	1605
v_asym_ (COO^−^)	1398	1433	1406	1452
v_sym_ (COO^−^)	1342	1382	1350	1305
v (N-O)	1348	1484, 1324	-	-

**Table 3:** Thermal analytical data for the complexes.

Complex	Molecular Formula	Stages	Temperature of TGA (°C)	Temperature of DTG (°C)	Mass Loss (%)		Assignment
					found	Calc.	
**3**	C_10_H_12_CuN_2_O_7_	I II III	79–192 192–320 320–484	138 243 392	12 30 37	11	2 H_2_O
**4**	C_44_H_44_Cu_4_N_4_O_12_	I II III	193–267 267–363 363–453	233 291 403	39 32 9	-	-

**Table 4:** Electronic spectral data (nm) of the ligands and their complexes.

Compound	π–π *	*n*–π *	d–d
**1**	241	382	
**3**	249	364	652
**2**	254	338	
**4**	267	377	638

**Table 5:** EPR spectral assignments of Cu (II) complexes in polycrystalline state at 298 K and solution at 77 K.

Compound	Polycrystalline State (298K)				Methanol Solution (77 K)					
	*g_iso_*	g_||_	*g*_┴_	G	g_||_	*g*_┴_	g_av_	A_||_ ^a^	A_┴_^a^	A_av_^a^
**3**	-	2.203	2.047	4.4899	2.271	2.065	2.1336	164	14	64
**4**	-	2.231	2.086	2.7323	2.266	2.053	2.1240	166	13	64

a: values in 10^−4^ cm^−1.^

**Table 6:** Catalytic cyclopropanation of styrene by EDA ^a^.

Complex	Conversion (%) ^b^	Yield (%) ^c^	*trans/cis* ^c^
**3**	>99.9	96	98/2
**4**	>99.9	90	>99.9/<0.1

^a^ Experimental conditions: EDA (1 mmoL) dissolved in C_2_H_4_Cl_2_ (2.8 mL) was slowly added (360 min) to a 25 °C solution of cat (0.05 mmoL) and styrene (8.7 mmoL) in dichloroethane (3 mL). ^b^ Conversion based on the starting EDA. ^c^ Determined by GC analysis: yield (%) = (ΣA_(*trans* + *cis*) cyclopropanes_/ΣA_(*trans* + *cis*) cyclopropanes + homocoupling products (fumarate + malate)_) × 100; *trans/cis*: A*_trans_*_cyclopropane_/A*_cis_*_cyclopropane_.

## Data Availability

Data is contained within the article.

## Supplementary Materials

The following are available online, Table S1. Crystal data and structure refinement of copper(II) complexes, Table S2. Fractional atomic coordinates (×10^4^) and equivalent isotropic displacement parameters (Å^2^ × 10^3^) for **3** (1). U_eq_ is defined as 1/3 of the trace of the orthogonalized U_IJ_ tensor, Table S3. Anisotropic displacement parameters (Å^2^ × 10^3^) for **3** (1). The anisotropic displacement factor exponent takes the form: −2π^2^ [h^2^a *^2^ U_11_+2hka * b * U_12_+…] Table S4. Bond lengths for **3** (1), Table S5. Bond angles for **3** (1), Table S6. Hydrogen atom coordinates (Å × 10^4^) and isotropic displacement parameters (Å2 × 10^3^) for **3** (1), Table S7. Atomic coordinates ( × 10^4^) and equivalent isotropic displacement parameters (Å2 × 10^3^) for **4** (2). U(eq) is defined as one-third of the trace of the orthogonalized Uij tensor, Table S8. Bond lengths (Å) for **4**. Table S9. Bond angles (°) for **4** (2). Table S9. Bond angles (°) for **4** (2). Table S10. Anisotropic displacement parameters (Å2 × 10^3^) for **4** (2). The anisotropic displacement factor exponent takes the form: −2p2 [ h2a * 2U11 + ... + 2 h k a * b * U12 ]. Figure S1. Infrared spectra of the ligand **1** and complex **3**, Figure S2. Infrared spectra of the ligand **2** and complex **4**, Figure S3. EPR spectra of complex **3** in polycrystalline at room temperature, Figure S4. EPR spectra of complex **4** in polycrystalline at room temperature, Figure S5. EPR spectra of complex **3** in frozen methanol at 77K, Figure S6. EPR spectra of complex **4** in frozen methanol at 77K.
