# Supplementary material for: Mononuclear and Tetranuclear Copper(II) Complexes Bearing Amino Acid Schiff Base Ligands: Structural Characterization and Catalytic Applications
Source: Molecules. 2021 Dec 1;26(23):7301. doi: 10.3390/molecules26237301 (PMC8658810; doi:10.3390/molecules26237301)
Supplement: Supplementary file 1 [file molecules-26-07301-s001.zip › molecules-1454598-supplementary.pdf]

Supporting information

Mononuclear and Tetranuclear Copper(II) Complexes Bearing Amino Acid Schiff Base Ligands: Structural Characterization and Catalytic Applications

Karla-Alejandra López-Gastélum [1,*], Enrique F. Velázquez-Contreras [1,*], Juventino J. García [2], Marcos Flores-Alamo [2], Gerardo Aguirre [3], Daniel Chávez-Velasco [3], Jayanthi Narayanan [4] and Fernando Rocha-Alonzo [5,*]

[1] Departamento de Investigación en Polímeros y Materiales, Universidad de Sonora, Rosales and Luis Encinas s/n, Col. Centro, Hermosillo, Sonora 83000, México

[2] Facultad de Química, Universidad Nacional Autónoma de México, Circuito Exterior Cd. Universitaria, Coyoacán, Ciudad México 04510, México; juvent@unam.mx (J.J.G.); mfa@unam.mx (M.F.-A.)

[3] Centro de Graduados e Investigación, Instituto Tecnológico de Tijuana, Apartado Postal 1166,
Tijuana Baja California 22000, Mexico; gaguirre777@gmail.com (G.A.); dchavez@hotmail.com (D.C.-V.)

[4] División de Ingeniería en Nanotecnología, Universidad Politécnica del Valle de México,
Av. Mexiquense s/n esquina Av. Universidad Politécnica, Tultitlan, Col. Villa Esmeralda, Tultitlan, Estado de México 54910, México; jnarayanan@upvm.edu.mx

[5] Departamento de Ciencias Químico Biológicas, Universidad de Sonora,
Calle Rosales y Blvd. Luis Encinas s/n, Col. Centro, Hermosillo, Sonora 83000, Mexico

* Correspondence: karla.lopezgastelum@gmail.com (K.-A.L.-G.); evlzqz@guaymas.uson.mx(E.F.V.-C.); fernando.rochaalonzo@unison.mx (F.R.A.)

Contents

Table S1. Crystal data and structure refinement of copper(II) complexes.
Table S2. Fractional Atomic Coordinates (×10^4) and Equivalent Isotropic Displacement Parameters (Å^2×10^3) for 3 (1). U_{eq} is defined as 1/3 of of the trace of the orthogonalized U_{IJ} tensor.
Table S3. Anisotropic Displacement Parameters (Å^2×10^3) for 3 (1). The Anisotropic displacement factor exponent takes the form: $-2\pi^2[h^2a^{*2}U_{11}+2hka^*b^*U_{12}+\ldots]$.
Table S4. Bond Lengths for 3 (1).
Table S5. Bond Angles for 3 (1).
Table S6. Hydrogen Atom Coordinates (Å×10^4) and Isotropic Displacement Parameters (Å^2×10^3) for 3 (1).
Table S7. Atomic coordinates (x 10^4) and equivalent isotropic displacement parameters (Å^{2}x 10^3) for 4 (2). U(eq) is defined as one third of the trace of the orthogonalized U_{ij} tensor.
Table S8. Bond lengths [Å] for 4.
Table S9. Bond angles [°] for 4 (2).
Table S10. Anisotropic displacement parameters (Å^{2}x 10^3) for 4 (2). The anisotropic displacement factor exponent takes the form: $-2p^2[\, h^2a^{*2}U^{11} + \ldots + 2\, h\, k\, a^*\, b^*\, U^{12}]$.
Figure S1. Infrared spectra of the ligand 1 and complex 3.
Figure S2. Infrared spectra of the ligand 2 and complex 4.
Figure S3. EPR spectra of complex 3 in polycrystalline at room temperature.
Figure S4. EPR spectra of complex 4 in polycrystalline at room temperature.
Figure S5. EPR spectra of complex 3 in frozen methanol at 77K.
Figure S6. EPR spectra of complex 4 in frozen methanol at 77K.

Table S1. Crystal data and structure refinement of copper(II) complexes.

	CuL_{NO2}	CuL_{CH3}
Empirical formula	$C_{10}H_{12}CuN_2O_7$	C_{44} H_{44} Cu_4 N_4 O_{12}
Formula weight	335.76	1074.99
Temperature/K	295.6 (2)	130(2)
Radiation	CuKα (λ =1.54184 Å)	MoKα (λ= 0.71073 Å)
Crystal system	Tetragonal	Tetragonal
Space group	I 41/a	I 41/a
a/ Å	14.8533(5)	22.4072(10)
b/ Å	14.8533(5)	22.4072(10)
c/ Å	22.2063(9)	8.4804(5)
α /°	90	90
β /°	90	90
γ /°	90	90
Volume/ $Å^3$	4899.2(4)	4257.9(5)
Z	16	4
Density (calculated)	1.821 g/cm^3	1.677 Mg/m^3
Absorption coefficient	2.891 mm^{-1}	2.041 mm^{-1}
F(000)	2736	2192
Crystal size	0.180 x 0.123 x 0.089 mm^3	0.560 x 0.150 x 0.140 mm^3
Theta range for data collection	7.160 to 152.832°	3.635 to 29.431°
Index ranges	-18 ≤ h ≤ 16, -18 ≤ k ≤ 18, -24 ≤ l ≤ 27	-30 ≤ h ≤ 19, -26 ≤ k ≤ 24, -11≤ l ≤ 8
Reflections collected	7354	5790
Independent reflections	2550 [R_{int} = 0.0265, R_{sigma} = 0.0240]	2543 [R_{int} = 0.0243]
Data / restraints / parameters	2550/0/186	2543/0/147
Goodness-of-fit on F^2	1.076	1.065
Final R indices [I>2σ(I)]	R_1 = 0.0476, wR_2 = 0.1318	R_1 = 0.0325, wR_2 = 0.0765
R indices (all data)	R_1 = 0.0564, wR_2 = 0.1394	R_1 = 0.0420, wR_2 = 0.0813
Largest diff. peak and hole	0.63 and -0.55 e.$Å^{-3}$	0.586 and -0.319 e.$Å^{-3}$

$R_1 = \sum||F_o| - |F_c||/\sum|F_o|$.

$wR_2 = [\sum w(F_o^2 - F_c^2)^2/\sum w(F_o^2)^2]^{1/2}$.

Table S2. Fractional Atomic Coordinates (×10^4) and Equivalent Isotropic Displacement Parameters ($Å^2$×10^3) for CuL_{NO2} (1). U_{eq} is defined as 1/3 of of the trace of the orthogonalized U_{IJ} tensor.

Atom	*x*	*y*	*z*	U(eq)
Cu1	5243.4(3)	8681.1(3)	3087.3(2)	36.90(19)
O1	6115.8(15)	8731.0(19)	3681.3(10)	47.0(6)
O2	4366.3(16)	8712.4(19)	2463.3(11)	49.1(6)
O3	2915.0(16)	8630(2)	2279.5(11)	54.3(7)
O4	5054(2)	9160(3)	6384.1(14)	91.1(13)
O5	6448.4(19)	9462.0(19)	6412.8(12)	54.7(6)
O6	6178.0(16)	8576(2)	2489.9(11)	49.8(6)
N1	4243.6(16)	8567.4(18)	3636.4(11)	35.5(5)
N2	5774(2)	9239(2)	6137.1(13)	48.7(7)
C1	5962(2)	8841(2)	4253.6(14)	38.8(7)
C2	6729(2)	9045(3)	4611.7(16)	46.1(8)
C3	6675(2)	9166(2)	5219.5(16)	43.8(7)
C4	5839(2)	9079(2)	5493.3(14)	40.6(7)
C5	5077(2)	8873(2)	5173.0(15)	40.1(7)
C6	5111(2)	8744(2)	4544.2(15)	36.0(6)
C7	4261(2)	8559(2)	4217.5(14)	36.6(6)
C8	3394(2)	8462(2)	3300.1(15)	40.4(7)
C9	3556(2)	8614(2)	2634.8(15)	41.7(7)
C10	3416(2)	8398(3)	4571.2(16)	47.6(8)
O7	5847(3)	8604(2)	1307.5(12)	67.7(8)

Table S3. Anisotropic Displacement Parameters ($Å^2×10^3$) for CuL_{NO2} (1). The Anisotropic displacement factor exponent takes the form: $-2π^2[h^2a^{*2}U_{11}+2hka^*b^*U_{12}+…]$.

Atom	U_{11}	U_{22}	U_{33}	U_{23}	U_{13}	U_{12}
Cu1	31.5(3)	49.3(3)	29.9(3)	0.61(18)	0.62(17)	-0.56(18)
O1	32.8(11)	76.3(17)	31.9(11)	-4.9(11)	1.6(9)	-0.7(10)
O2	35.1(12)	79.7(18)	32.4(11)	2.5(11)	-3.0(9)	-0.6(11)
O3	35.2(12)	88(2)	39.8(13)	5.0(13)	-6(1)	0.4(12)
O4	58.9(19)	179(4)	35.3(15)	-8(2)	5.1(14)	-24(2)
O5	59.9(16)	64.5(17)	39.6(13)	-5.8(12)	-10.2(12)	-2.0(12)
O6	37.4(12)	80.8(18)	31.3(11)	-1.1(12)	1.0(9)	0.2(11)
N1	30.3(12)	45.1(14)	31.0(13)	0.5(10)	-0.6(10)	1.4(10)
N2	52.0(17)	60.0(18)	34.1(15)	1.0(13)	-3.8(13)	0.3(14)
C1	36.0(15)	47.7(17)	32.9(15)	1.7(13)	-2.3(12)	2.5(13)
C2	36.7(16)	62(2)	39.3(17)	-0.1(16)	1.2(14)	-3.1(14)
C3	40.9(17)	50.4(18)	40.0(17)	-1.7(14)	-6.6(14)	0.8(13)
C4	46.7(17)	44.1(17)	31.1(16)	0.8(13)	-3.1(13)	2.8(13)
C5	39.1(16)	46.7(17)	34.7(16)	3.7(13)	0.3(13)	0.5(13)
C6	36.3(15)	39.6(15)	32.1(15)	2.5(12)	0.6(12)	-0.2(11)
C7	35.4(15)	39.3(16)	35.2(15)	-0.7(12)	1.1(13)	0.8(12)
C8	28.6(14)	55.5(19)	37.1(16)	-1.8(14)	-0.9(12)	-0.5(13)
C9	37.0(16)	53.0(19)	35.0(16)	0.9(14)	-1.8(13)	0.1(13)
C10	39.6(17)	66(2)	36.9(17)	-0.1(16)	4.2(14)	-7.1(15)
O7	93(2)	75(2)	35.0(14)	-1.4(13)	3.2(15)	0.3(18)

Table S4. Bond Lengths for CuL_{NO2} (1).

Atom	Atom	Length/Å	Atom	Atom	Length/Å
Cu1	O1	1.851(2)	N2	C4	1.452(4)
Cu1	O2	1.902(2)	C1	C2	1.422(5)
Cu1	O6	1.927(2)	C1	C6	1.427(4)
Cu1	N1	1.929(3)	C2	C3	1.364(5)
O1	C1	1.301(4)	C3	C4	1.388(5)
O2	C9	1.271(4)	C4	C5	1.372(5)
O3	C9	1.237(4)	C5	C6	1.410(5)
O4	N2	1.208(4)	C6	C7	1.482(4)
O5	N2	1.220(4)	C7	C10	1.499(4)
N1	C7	1.291(4)	C8	C9	1.514(4)
N1	C8	1.475(4)			

Table S5. Bond Angles for CuL_{NO2} (1).

Atom	Atom	Atom	Angle/˚	Atom	Atom	Atom	Angle/˚
O1	Cu1	O2	176.10(12)	C3	C2	C1	122.3(3)
O1	Cu1	O6	89.40(10)	C2	C3	C4	118.3(3)
O1	Cu1	N1	95.28(11)	C3	C4	N2	118.4(3)
O2	Cu1	O6	89.64(11)	C5	C4	N2	119.5(3)
O2	Cu1	N1	86.30(11)	C5	C4	C3	122.1(3)
O6	Cu1	N1	169.41(12)	C4	C5	C6	121.0(3)
C1	O1	Cu1	125.3(2)	C1	C6	C7	123.5(3)
C9	O2	Cu1	115.3(2)	C5	C6	C1	117.7(3)
C7	N1	Cu1	128.2(2)	C5	C6	C7	118.7(3)
C7	N1	C8	121.5(3)	N1	C7	C6	120.3(3)
C8	N1	Cu1	110.36(19)	N1	C7	C10	120.6(3)
O4	N2	O5	121.7(3)	C6	C7	C10	119.1(3)
O4	N2	C4	119.4(3)	N1	C8	C9	110.0(3)
O5	N2	C4	118.9(3)	O2	C9	C8	117.4(3)
O1	C1	C2	115.6(3)	O3	C9	O2	122.4(3)

O1	C1	C6	125.8(3)	O3	C9	C8	120.2(3)
C2	C1	C6	118.6(3)				

Table S6. Hydrogen Atom Coordinates (Å×10[4]) and Isotropic Displacement Parameters (Å[2]×10[3]) for CuL_{NO2} (1).

Atom	*x*	*y*	*z*	U(eq)
H6A	5941.7	8531.47	2141.78	75
H6B	6491.11	8108.53	2561.63	75
H2	7286.97	9098.06	4424.96	55
H3	7184.35	9304.27	5444.58	53
H5	4530.31	8817.76	5373.41	48
H8A	2954.43	8892.97	3446.58	49
H8B	3155.25	7861.9	3363.59	49
H10A	3162.16	8965.19	4690.63	71
H10B	3554.23	8048.95	4923.04	71
H10C	2990.48	8077.4	4326.53	71
H7A	5999.71	8941.38	1015.33	102
H7B	5934.07	8056.97	1212.52	102

Table S7. Atomic coordinates (x 10[4]) and equivalent isotropic displacement parameters (Å[2]x 10[3]) for CuL_{CH3} (2). U(eq) is defined as one third of the trace of the orthogonalized U^{ij} tensor.

Atom	x	y	z	U(eq)
C(1)	-903(1)	1659(1)	-2509(2)	21(1)
C(2)	-849(1)	1421(1)	-844(3)	23(1)
C(3)	-144(1)	850(1)	675(2)	18(1)
C(4)	422(1)	532(1)	887(3)	19(1)
C(5)	863(1)	477(1)	-315(3)	20(1)
C(6)	1404(1)	190(1)	73(3)	26(1)
C(7)	1511(1)	-36(1)	1551(3)	27(1)
C(8)	1077(1)	-1(1)	2741(3)	24(1)
C(9)	549(1)	276(1)	2378(3)	21(1)
C(10)	1193(1)	-245(1)	4375(3)	31(1)
C(11)	-559(1)	891(1)	2074(3)	28(1)
Cu(1)	-1397(1)	2324(1)	-4914(1)	19(1)
N(1)	-283(1)	1097(1)	-661(2)	19(1)
O(1)	-483(1)	1560(1)	-3472(2)	32(1)
O(2)	-1362(1)	1948(1)	-2833(2)	20(1)
O(3)	809(1)	666(1)	-1778(2)	24(1)

Table S8. Bond lengths [Å] for CuL_{CH3}.

Atoms	Lengths/Å	Atoms	Lengths/Å
C(1)-O(2)	1.246(3)	C(7)-H(7)	0.9500
C(1)-O(1)	1.266(3)	C(8)-C(9)	1.372(3)
C(1)-C(2)	1.514(3)	C(8)-C(10)	1.512(3)
C(2)-N(1)	1.471(3)	C(9)-H(9)	0.9500
C(2)-H(2A)	0.9900	C(10)-H(10A)	0.9800
C(2)-H(2B)	0.9900	C(10)-H(10B)	0.9800
C(3)-N(1)	1.298(3)	C(10)-H(10C)	0.9800
C(3)-C(4)	1.467(3)	C(11)-H(11A)	0.9800
C(3)-C(11)	1.510(3)	C(11)-H(11B)	0.9800
C(4)-C(9)	1.416(3)	C(11)-H(11C)	0.9800
C(4)-C(5)	1.426(3)	Cu(1)-O(3)#1	1.8560(17)

C(5)-O(3)	1.316(3)	Cu(1)-N(1)#1	1.9288(18)
C(5)-C(6)	1.412(3)	Cu(1)-O(1)#1	1.9467(17)
C(6)-C(7)	1.372(3)	Cu(1)-O(2)	1.9572(15)
C(6)-H(6)	0.9500	C(7)-H(7)	0.9500
C(7)-C(8)	1.403(4)	C(8)-C(9)	1.372(3)

Table S9. Bond angles [°] for CuL_{CH3} (2).

Atoms	Angles/°	Atoms	Angles/°
O(2)-C(1)-O(1)	124.2(2)	C(6)-C(7)-C(8)	121.0(2)
O(2)-C(1)-C(2)	117.0(2)	C(6)-C(7)-H(7)	119.5
O(1)-C(1)-C(2)	118.7(2)	C(8)-C(7)-H(7)	119.5
N(1)-C(2)-C(1)	109.98(18)	C(9)-C(8)-C(7)	117.5(2)
N(1)-C(2)-H(2A)	109.7	C(9)-C(8)-C(10)	121.1(2)
C(1)-C(2)-H(2A)	109.7	C(7)-C(8)-C(10)	121.3(2)
N(1)-C(2)-H(2B)	109.7	C(8)-C(9)-C(4)	123.8(2)
C(1)-C(2)-H(2B)	109.7	C(8)-C(9)-H(9)	118.1
H(2A)-C(2)-H(2B)	108.2	C(4)-C(9)-H(9)	118.1
N(1)-C(3)-C(4)	121.4(2)	C(8)-C(10)-H(10A)	109.5
N(1)-C(3)-C(11)	120.9(2)	C(8)-C(10)-H(10B)	109.5
C(4)-C(3)-C(11)	117.78(19)	H(10A)-C(10)-H(10B)	109.5
C(9)-C(4)-C(5)	117.7(2)	C(8)-C(10)-H(10C)	109.5
C(9)-C(4)-C(3)	118.7(2)	H(10A)-C(10)-H(10C)	109.5
C(5)-C(4)-C(3)	123.64(19)	H(10B)-C(10)-H(10C)	109.5
O(3)-C(5)-C(6)	116.4(2)	C(3)-C(11)-H(11A)	109.5
O(3)-C(5)-C(4)	125.7(2)	C(3)-C(11)-H(11B)	109.5
C(6)-C(5)-C(4)	117.9(2)	H(11A)-C(11)-H(11B)	109.5
C(7)-C(6)-C(5)	122.1(2)	C(3)-C(11)-H(11C)	109.5
C(7)-C(6)-H(6)	119.0	H(11A)-C(11)-H(11C)	109.5
C(5)-C(6)-H(6)	119.0	H(11B)-C(11)-H(11C)	109.5
C(6)-C(7)-C(8)	121.0(2)	O(3)#1-Cu(1)-N(1)#1	95.19(7)
C(6)-C(7)-H(7)	119.5	O(3)#1-Cu(1)-O(1)#1	178.97(7)
C(8)-C(7)-H(7)	119.5	N(1)#1-Cu(1)-O(1)#1	85.82(7)
C(9)-C(8)-C(7)	117.5(2)	O(3)#1-Cu(1)-O(2)	91.42(7)
C(9)-C(8)-C(10)	121.1(2)	N(1)#1-Cu(1)-O(2)	173.12(7)
C(7)-C(8)-C(10)	121.3(2)	O(1)#1-Cu(1)-O(2)	87.56(7)
O(3)-C(5)-C(6)	116.4(2)	C(3)-N(1)-C(2)	120.55(19)
O(3)-C(5)-C(4)	125.7(2)	C(3)-N(1)-Cu(1)#2	127.97(16)
C(6)-C(5)-C(4)	117.9(2)	C(2)-N(1)-Cu(1)#2	111.48(13)
C(7)-C(6)-C(5)	122.1(2)	C(1)-O(1)-Cu(1)#2	114.00(14)
C(7)-C(6)-H(6)	119.0	C(1)-O(2)-Cu(1)	117.13(14)
C(5)-C(6)-H(6)	119.0	C(5)-O(3)-Cu(1)#2	126.02(15)

Symmetry transformations used to generate equivalent atoms:
#1 y-1/4,-x+1/4,-z-3/4 #2 -y+1/4,x+1/4,-z-3/4

Table S10. Anisotropic displacement parameters ($Å^2$x 10^3)for CuL_{CH3} (2). The anisotropic displacement factor exponent takes the form: $-2p^2[h^2a^{*2}U^{11} + ... + 2 h k a^* b^* U^{12}]$.

	U^{11}	U^{22}	U^{33}	U^{23}	U^{13}	U^{12}
C(1)	27(1)	23(1)	13(1)	1(1)	1(1)	0(1)
C(2)	26(1)	28(1)	14(1)	5(1)	4(1)	7(1)
C(3)	25(1)	16(1)	13(1)	1(1)	1(1)	-2(1)
C(4)	25(1)	17(1)	15(1)	-1(1)	0(1)	-1(1)
C(5)	28(1)	19(1)	15(1)	3(1)	2(1)	0(1)
C(6)	24(1)	31(1)	23(1)	3(1)	2(1)	3(1)
C(7)	28(1)	26(1)	26(1)	3(1)	-4(1)	5(1)
C(8)	36(1)	19(1)	17(1)	1(1)	-2(1)	0(1)
C(9)	29(1)	19(1)	14(1)	-1(1)	0(1)	0(1)
C(10)	42(2)	31(1)	20(1)	7(1)	-4(1)	7(1)
C(11)	31(1)	39(2)	14(1)	7(1)	5(1)	9(1)
Cu(1)	21(1)	25(1)	10(1)	3(1)	-3(1)	-2(1)

N(1)	23(1)	21(1)	13(1)	3(1)	2(1)	3(1)
O(1)	35(1)	48(1)	12(1)	10(1)	6(1)	17(1)
O(2)	23(1)	25(1)	13(1)	4(1)	-1(1)	2(1)
O(3)	26(1)	31(1)	16(1)	7(1)	4(1)	5(1)

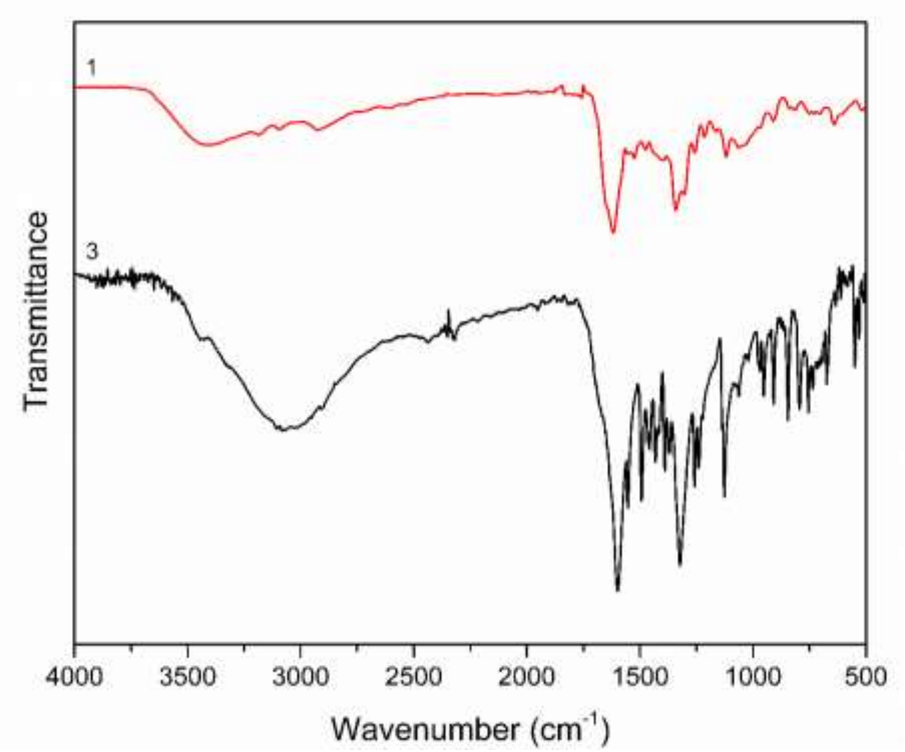

Figure S1. Infrared spectra of the ligand 1 and complex 3.

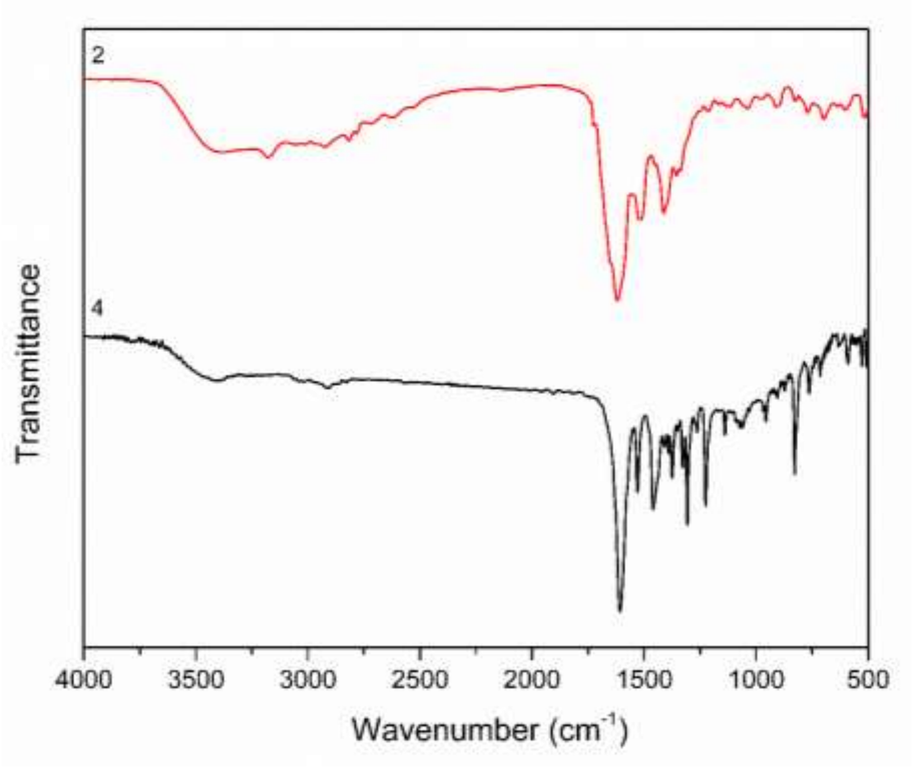

Figure S2. Infrared spectra of the ligand 2 and complex 4.

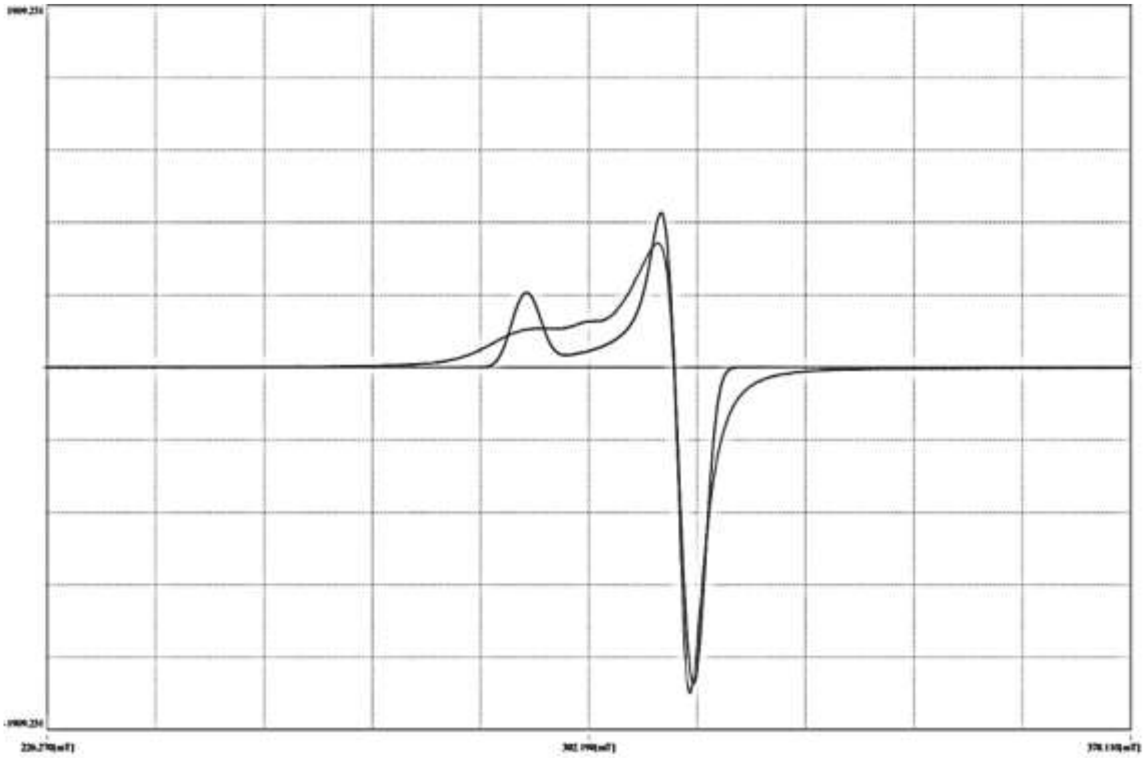

Figure S3. EPR spectra of complex 3 in polycrystalline at room temperature.

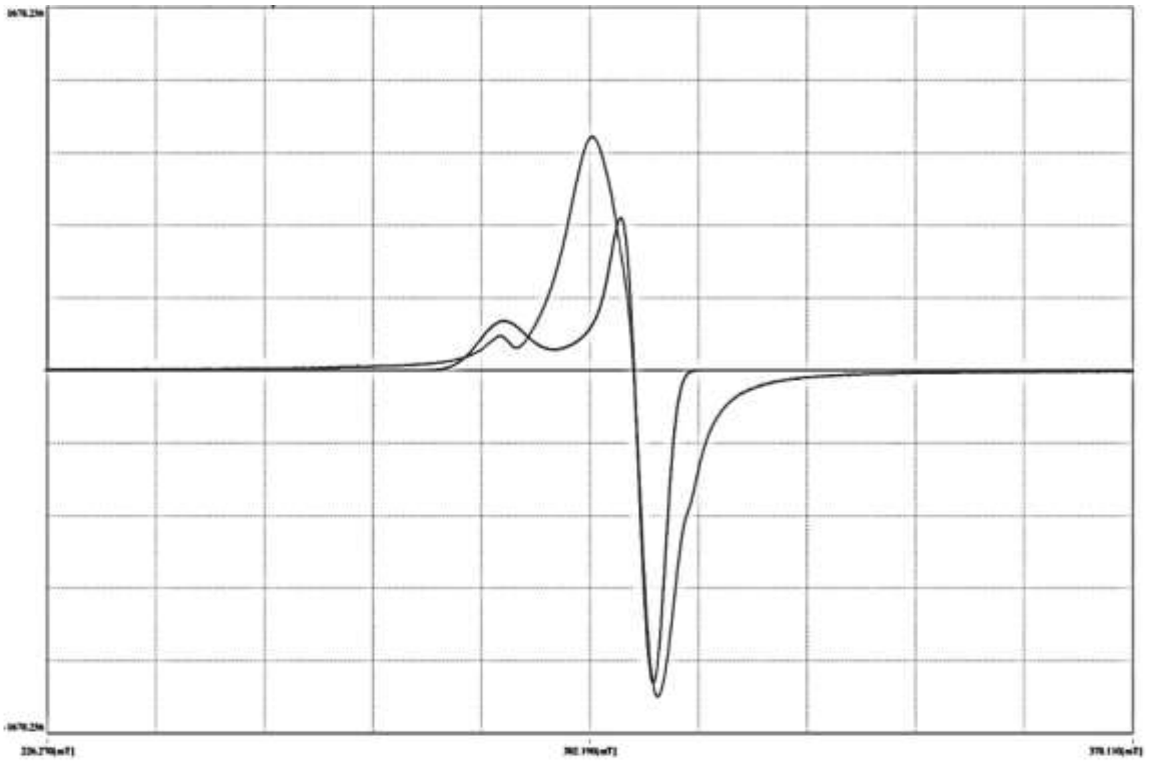

Figure S4. EPR spectra of complex 4 in polycrystalline at room temperature.

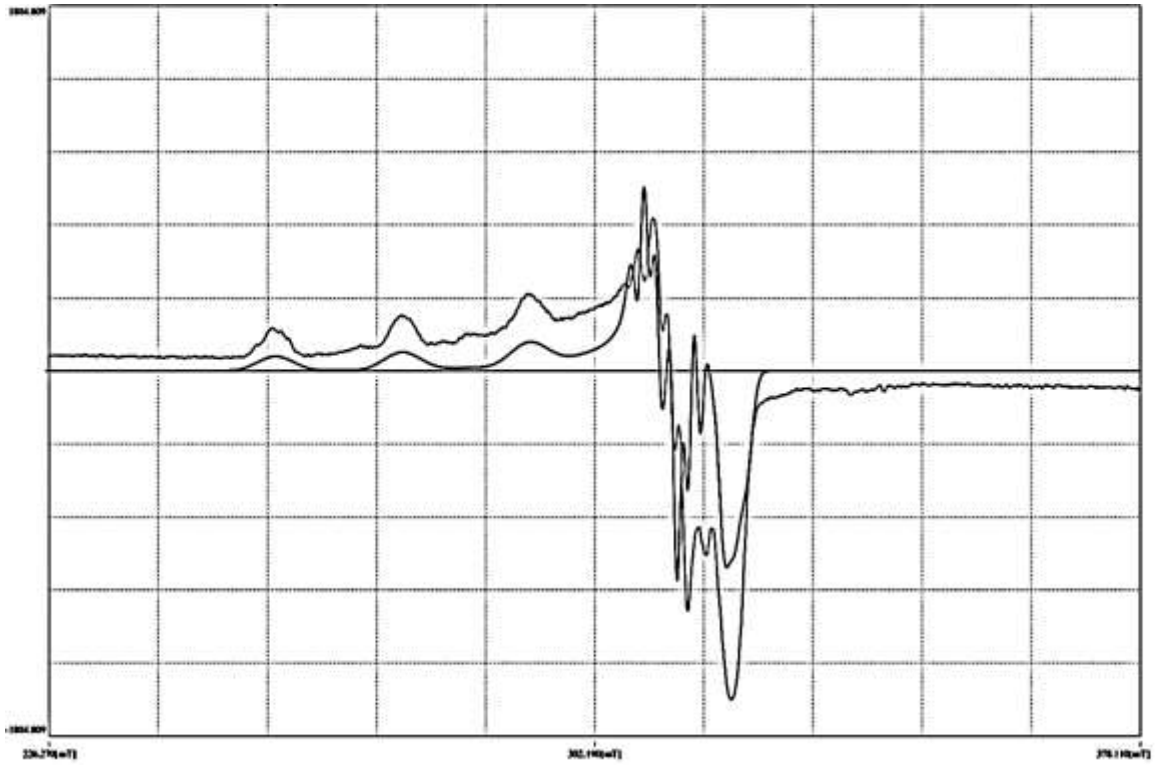

Figure S5. EPR spectra of complex 3 in frozen methanol at 77K.

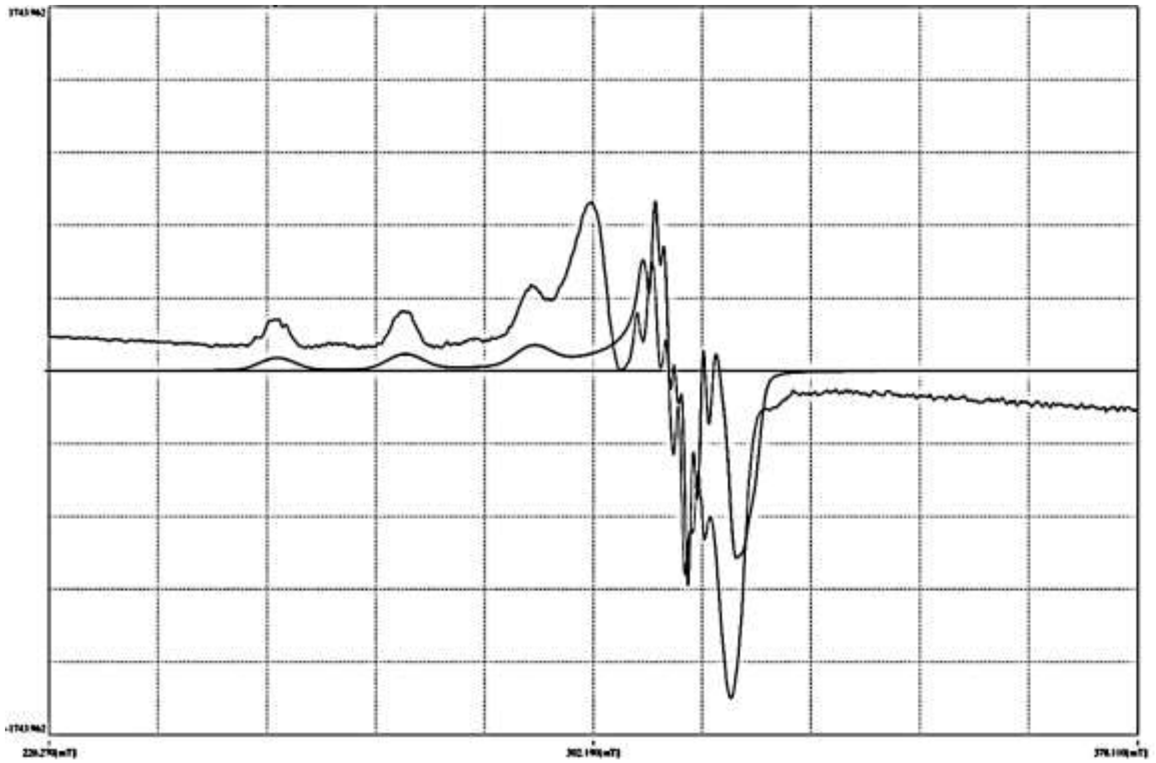

Figure S6. EPR spectra of complex 4 in frozen methanol at 77K.
